# Next-Generation Sequencing of the Complete Huaibei Grey Donkey Mitogenome and Mitogenomic Phylogeny of the *Equidae* Family

**DOI:** 10.3390/ani13030531

**Published:** 2023-02-02

**Authors:** Jingjing Xia, Liang Chang, Dashuang Xu, Yuqing Jia, Yuanfei Ding, Chengcheng Cao, Zhaoyu Geng, Sihua Jin

**Affiliations:** 1 College of Animal Science and Technology, Anhui Agricultural University, Hefei 230036, China; 2 Anhui Livestock and Poultry Genetic Resources Protection Center, Hefei 231283, China

**Keywords:** huaibei grey donkey, mitogenome, D-loop, phylogenetic, genetic diversity, maternal origin

## Abstract

**Simple Summary:**

The phylogenetic status of the Huaibei grey donkey based on its complete mitochondrial genome, phylogeny, and maternal origin has not been fully established. This study reports the mitochondrial DNA diversity of the Huaibei grey donkey breed and presents its origin and genetic characterization. The Huaibei grey donkey’s complete mitogenome was 16,680 bp in length, containing 22 tRNAs, 2 rRNAs, 13 PCGs, and 1 D-loop region. The median-joining network and phylogenetic tree indicated two possible maternal lineages, with the Somali lineage as the most probable domestication center of Huaibei grey donkey. These results provide novel information on the origin and phylogeny of the Huaibei grey donkey and can be used as a reference for breeding and conservation management.

**Abstract:**

The Huaibei grey donkey (HGD) is an endangered species and a vital native breed in Anhui Province, China. However, its complete mitogenome, phylogeny, and maternal origin remain unclear. The objectives of this study were to detect the genetic diversity of the HGD and investigate its phylogenetic relationship with other breeds to inform conservation management. The complete mitogenome of the HGD was sequenced through next-generation sequencing, and the most variable region in the mitochondrial DNA displacement-loop (D-loop) was amplified via a polymerase chain reaction (PCR). Next, we used the median-joining network (MJN) to calculate the genetic relationships among populations and the neighbor-jointing method to build a phylogenetic tree and speculate as to its origin. The results showed that the mitogenome contains 22 tRNAs, 2 rRNAs, 13 PCGs, and 1 D-loop region. Analyzing the D-loop region of the HGDs, we identified 23 polymorphic sites and 11 haplotypes. The haplotype and nucleotide diversity were 0.87000 (Hd) and 0.02115 (Pi), respectively. The MJN analysis indicated that the HGD potentially has two maternal lineages, and phylogenetic analysis indicated that the Somali lineage could be the most probable domestication center for this breed. Therefore, our mitogenome analysis highlights the high genetic diversity of the HGD, which may have originated from the Somali wild ass, as opposed to the Asian wild ass. This study will provide a useful resource for HGD conservation and breeding.

## 1. Introduction

Mitochondrial DNA (mtDNA) is an extranuclear genetic material with a specific structure that does not undergo recombination during generational transmission and is maternally derived [1,2]. As a genetic marker, mtDNA is highly significant in genetic and evolutionary livestock studies [3,4]. mtDNA contains exons and a non-coding region—the control region (CR)—also known as the displacement-loop region (D-loop) [5]. In the mammalian mtDNA D-loop, the most variable region exists between tRNA^Pro^ and the large conserved sequence block [6]. Additionally, mtDNA has been used to infer wild ancestors, determine the domestication centers of modern domestic animals, and study several domestic animals’ origins, evolutionary relationships, and genetic diversity [3,7].

Donkey domestication is generally considered to have taken place in the tropics or subtropics of Africa [8]; however, this remains unclear. Studies of donkey mitochondrial sequences have suggested that there were two highly differentiated maternal lineages (Clade I and Clade II) during donkey (*Equus asinus*) domestication. Clade I clusters clearly with the Nubian wild ass (*Equus africanus africanus*), and Clade II originates from the Somali wild ass (*E. africanus somaliensis*), which is likely nearing extinction [8,9,10]. In recent years, an increasing number of studies on donkey mtDNA have assessed evolutionary relationship, variation, and genetic diversity among Asian, European, and African breeds [11,12,13,14,15,16].

Chinese donkeys are categorized according to body size: small, medium, and large. The Huaibei grey donkey (HGD) is a small-sized donkey with grey fur, and their main production area is in Huaibei City, Anhui Province. It is considered an important local breed in China, which has gained more attention owing to its strong disease resistance, coarse feed tolerance, and better adaptability. However, few studies have analyzed the complete mitochondrial genome, genetic diversity, and maternal origin of HGDs. Therefore, we used next-generation sequencing and normal polymerase chain reaction (PCR) sequencing methods to analyze the genetic diversity and detect the partial sequence polymorphisms of the D-loop and the evolutionary relationships of HGDs to inform their management.

## 2. Materials and Methods

### 2.1. Ethical Statement

**Figure 1:**
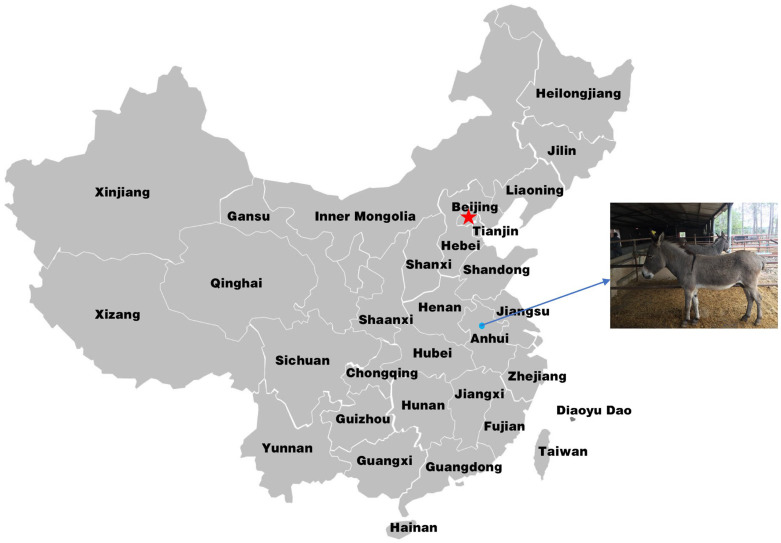
Geographic location of the Huaibei grey donkey.

The experimental donkeys were obtained from Anhui Domestic Donkey Conservation and Breeding Co., Ltd., Anhui, China (Figure 1). All works were conducted in accordance with and approval from the Animal Care and Use Committee of Anhui Agricultural University (approval ID: SYXK 2016-007).

### 2.2. Specimen Collection and DNA Isolation

Samples were collected from Anhui Domestic Donkey Conservation and Breeding Co., Ltd., Anhui, China (Figure 1). The total genomic DNA was isolated using a blood DNA extraction kit (Tiangen Biotech Co., Ltd., Beijing, China) and stored at −20 °C. DNA integrity was evaluated via 1.5% agarose gel electrophoresis. The most variable region in the mtDNA D-loop of 60 samples was amplified using PCR. One female HGD was selected at random to determine the complete mitochondrial genome sequence.

### 2.3. Amplification of Part of the mtDNA D-Loop and Sequencing

The proximal sequences of the D-loop region (418 bp, between 15,419 bp to 15,836 bp, GenBank accession number NC_001788.1 [17]) of the *trnP* gene and the central conserved sequence block [18] were amplified by PCR using appropriate primers (F: 5′-ACCACTCGCAAGCACCA-3′; R: 5′-CACAGCATCCCCAAATA-3′). The primers were designed in Primer 3 [19] and synthesized and purified by TSINGKE Biotechnology Co., Ltd. (Nanjing, China). PCR amplification was performed in a 50 μL system containing 2 μL of template DNA, 25 μL 2× of Taq Master Mix, 2 μL of each primer, and 19 μL of ddH_2_O. The amplification conditions were as follows: 94 °C for 5 min, 35 cycles at 94 °C for 30 s, 55 °C for 30 s, 72 °C for 30 s, and a final extension step at 72 °C for 10 min. The complete mitogenome was obtained via whole-genome shotgun sequencing using an Illumina NovaSeq 6000 platform (Illumina, Inc., San Diego, CA, USA) with paired-end read lengths of 150 bp.

### 2.4. Sequence Assembly Annotation and Analysis

After quality control, Bowtie2 was used to align clean the data from the complete mtDNA of the HGDs against a reference genome (GenBank, NC_001788.1). The reads on the alignment were retained, and SPAdes 3.13.0 (parameters: K 127) was used for genome assembly. The splicing results were compared with the circular reference genome using BLASTN, with the assembly results determined accordingly. tRNAs were recognized and their secondary structures predicted using tRNAscan-SE v2.0 [20]. We derived a circular map of the mitogenome using ORDRAW [21], and the base composition skew was calculated using the formulas AT-skew = (A − T)/(A + T) and GC-skew = (G − C)/(G + C) [22]. The most variable region was identified through sequence alignment against the reference genome. The 418 bp fragments were edited manually using Contig Express software (Contig Express LLC, New York, NY, USA) and aligned with the reference sequence using CLUSTALX 2.0 [23]. DNAsp v6 [24] was used to calculate the nucleotide diversity, number of polymorphic sites, and haplotype diversity of the most variable region in the HGD mtDNA D-loop region.

### 2.5. Phylogenetic Analysis

The genetic relationships among populations were determined based on the MJN using Network v.10.1.0.0 software [25]. The MJN was constructed from 11 haplotype sequences discovered in this research and 60 reference sequences found previously [15,26]; 15 sequences—Hap4, Hap6, Hap7, and Hap10, Hap12, Hap16, Hap18, Hap20, Hap22, Hap23, Hap24, Hap25, Hap26, Hap27, and Hap29—belong to Clade I. Reference mtDNA D-loop region sequences of wild and domestic donkeys were obtained from GenBank and used as comparators of the HGD to build a phylogenetic tree and speculate about their origin [9,11,15,16,27,28]. The phylogenetic tree was drawn using the neighbor-joining method based on the Kimura-2-parameter genetic distances [29], and bootstrap values were estimated using 1000 repetitions [30] and reconstructed using MEGA v7.0 [31].

## 3. Results

### 3.1. Structure and Organization of the Complete Mitochondrial Genome

**Figure 2:**
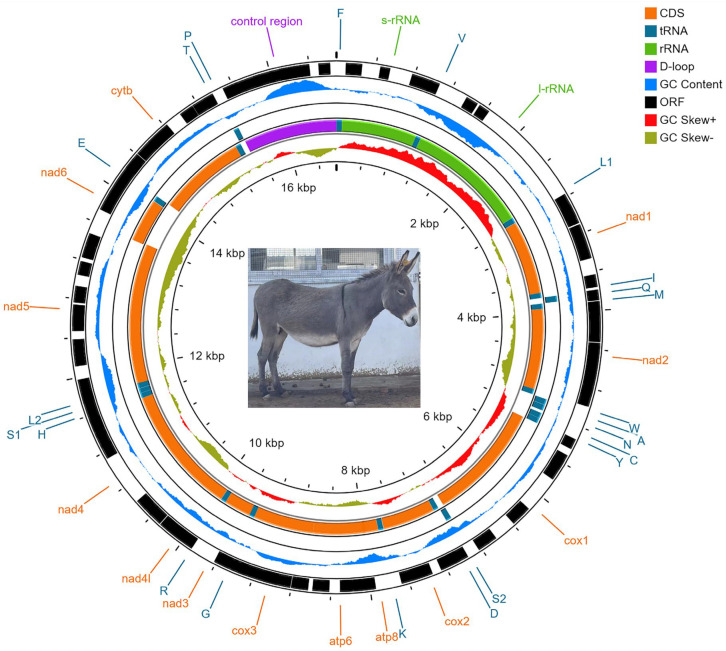
Gene map of the Huaibei grey donkey. Note: W: tRNA-Trp, A: tRNA-Ala, N: tRNA-Asn, Y: tRNA-Tyr, C: tRNA-Cys, D: tRNA-Asp, G: tRNA-Gly, K: tRNA-Lys, R: tRNA-Arg, H: tRNA-His, S1: tRNA-Ser1, L2: tRNA-Leu2, E: tRNA-Glu, T: tRNA-Thr, P: tRNA-Pro, F: tRNA-Phe, V: tRNA-Val.

We deposited the complete mtDNA—a circular 16,680 bp molecule—in GenBank under accession no.MZ911746, displaying a characteristic circular structure of the genetic map (Figure 2). There are 13 protein coding genes (PCGs), including those encoding seven NADH dehydrogenase complex subunits (*ND1-6* and *ND4L*), three cytochrome oxidase subunits (*COX1-3*), two ATPase subunits (*ATP6* and *ATP8*), and cytochrome b (*CYTB*). Additionally, the mitogenome contained 2 rRNA genes (*rRNAL* and *rRNAS*), 22 tRNA genes, and 1 control region (D-loop). Regarding their location, 14 tRNA genes, 12 PCGs and 2 rRNA genes were located on the heavy strand, and the remaining 8 tRNA genes and *ND6* were on the light strand (Table 1). A bias in nucleotide composition was observed toward A (32.31%) and T (25.59%), with respect to C (28.88%) and G (13.22%). We observed a negative GC-skew (–0.3720) and a positive AT-skew (+0.1161), indicating that A and C were marginally more numerous than T and G (Table 2).

### 3.2. Codon Usage and Protein Coding Genes

**Figure 3:**
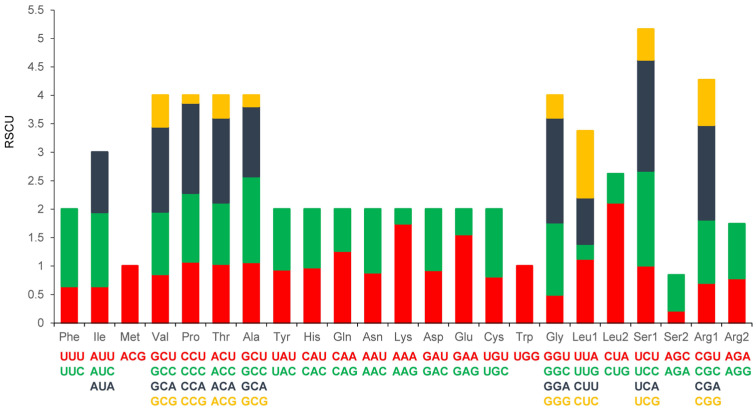
Relative synonymous codon usage (RSCU) in the Huaibei grey donkey mitochondrial genome.

The 13 PCGs had a full length of 9 911 bp comprising 57.3% AT nucleotides, with a positive AT-skew (+0.0750) and a negative GC-skew (–0.4239). Codons encoding Trp were infrequent, whereas those encoding Leu and Ser occurred most frequently (Figure 3). The PCG regions comprised a total of 5 559 codons. The initiation and termination signals, in addition to the gene lengths, are listed in Table 2. Eleven PCGs have ATG as their start codon, except for *ND2* and *ND3*, which have ATA as their start codon. Eight PCGs (*ND1*, *COX1*, *COX2*, *ATPase8*, *ATPase6*, *ND4L*, *ND5*, and *ND6*) had TAA as their stop codon; *ND2*, *COX3*, and *ND3* had TAG as their stop codon; and *ND4* and *CYTB* had AGA as their stop codon. The most frequently used amino acids in the PCGs of the HGD mitogenomes were Leu and Ser (12.4% and 12.4%, respectively); among the 64 available codons, the 3 most frequently used codons are CUA (2.12%) for Leu2, UCA (3.18%) for Ser1, and GGA (2.41%) for Gly. The relative synonymous codon usage (RSCU) values for HGDs are shown in Table 3.

### 3.3. Transfer and Ribosomal RNA Genes

**Figure 4:**
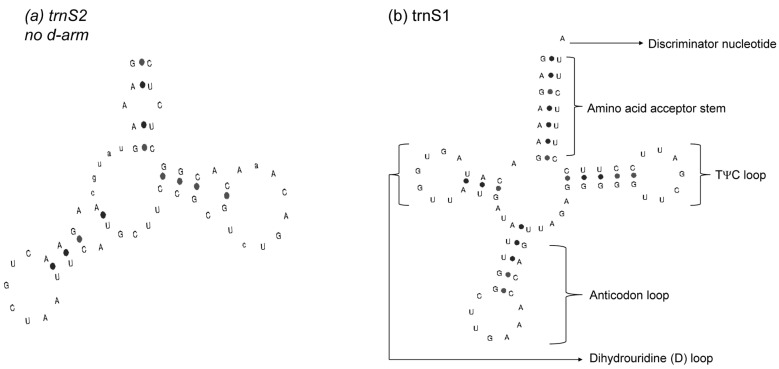
Secondary structure of tRNA-Ser.

The 22 tRNAs had a full length of 1 516 bp and 61.7% AT nucleotides, with a positive AT-skew (+0.1183) and a negative GC-skew (–0.1854). Most tRNA genes had the typical cloverleaf secondary structure, except for *tRNA*^Ser^ (GCT) (Figure 4). The full length of the two rRNAs was 2 555 bp, the AT content was 60.1%, the AT-skew was positive (+0.2164), and the GC-skew was negative (–0.1508). With respect to their location, *rrnL* was located between *trnV* and *trnL1*, and *rrnS* was between *trnV* and *trnF*. The lengths of *rrnL* and *rrnS* were 1 580 bp and 975 bp, respectively.

### 3.4. Genetic Variation and Genetic Diversity of Complete D-Loop Region

There were 23 polymorphic sites and 11 haplotypes in the mtDNA D-loop sequences (H1–H11, GenBank accession numbers: OP095367~OP095377). The AT content of the D-loop region was 52%, with a positive AT-skew (+0.1692) and a negative GC-skew (–0.4542) that was identified between *trnP* and *trnF*. The haplotype and nucleotide diversity values were 0.87000 (Hd) and 0.02115 (Pi), respectively. There are 5 singleton variable sites and 18 parsimony informative sites. All of the polymorphisms were A/G and T/C transitions, except at the site of 297. Our molecular studies of donkey mitochondrial sequences have defined two distinct matriarchal lineages (Clade I and II) related to domestication. We observe several polymorphic sites in Clade Ⅱ (66 A/G, 72 C/T, 85 C/T, 151 A/G, 162 A/G, 172 A/G, 174 A/G, 180 T/C, 202 T/C, 203 A/G, 226 A/G, 227 A/G, 234 T/C, 224 A/G, 280 T/C, 352 T/C, 383 T/C, 388 T/C, 402 T/C, and 404 A/G), but only three in Clade I (181 A/G, 297 A/T, and 403 A/G). The calculated number of polymorphic sites and the haplotypic diversity of HGDs are shown in Table 4.

### 3.5. Maternal Origin of Huaibei Grey Donkey

**Figure 5:**
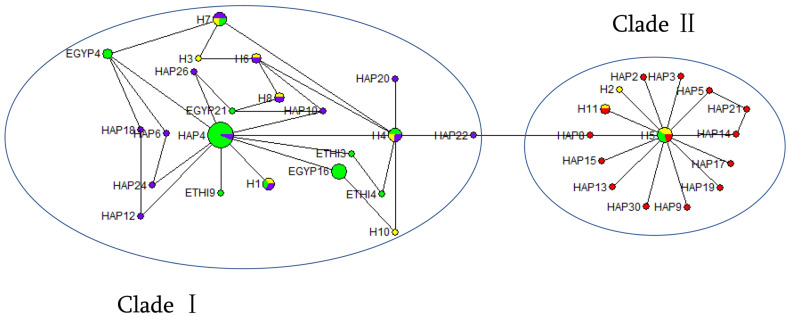
Median-joining network constructed from 11 haplotypes obtained from Huaibei grey donkey and 60 reference sequences obtained from 5 different populations. Note: The circle areas correspond to the haplotype frequency. Yellow circles indicate the haplotypes in this study. The green circles are 30 previously identified haplotypes [26]. The reference haplotypes Hap1~Hap30 were downloaded from NCBI [15], and the purple circles represent haplotypes belonging to Clade I (Hap4, Hap6, Hap7, Hap10, Hap12, Hap16, Hap18, Hap20, Hap22, Hap23, Hap24, Hap25, Hap26, Hap27 and Hap29), while the red circles represent Clade II haplotypes.

The 60 reference mtDNA D-loop sequences (Table 5) and HGD population sequences of 418 bp were compared to determine the relationships among the haplotypes and the population structure of HGDs. The MJN was constructed for the identified haplotypes. There were two distinct lineages (Clade I and Clade II) revealed by the MJN, and most HGDs were classified into Clade I (31 individuals (51.67%) and 4 haplotypes). Clade II included 29 individuals (48.33%) from 7 haplotypes (Figure 5).

**Figure 6:**
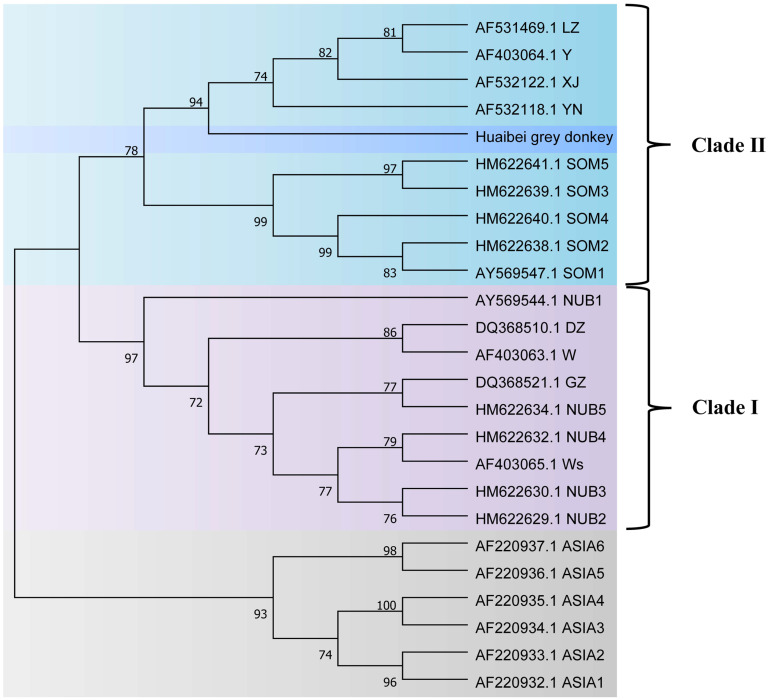
Phylogenetic relationships of Huaibei grey donkey determined using concatenated nucleotide sequences. Note: ASIA: Asian wild ass (*E. hemionus*), NUB: Nubian wild ass (*E. africanus africanus*), SOM: Somali wild ass (*E. africanus somaliensis*), LZ: Liangzhou donkey, XJ: Xinjiang donkey, YN: Yunnan donkey, DZ: Dezhou donkey, GZ: Guanzhong donkey, haplotype of Y, W, and Ws belong to European domestic donkeys.

For additional clarification of the HGD’s origin, we performed comparisons of the HGD mtDNA sequence with that of the Nubian wild ass (*E. africanus africanus*), Somali wild ass (*E. africanus somaliensis*), Asian wild ass (*E. hemionus*), and European and Chinese domestic donkeys. The HGDs were markedly clustered with the Somali wild ass sequences. Further, the phylogenetic tree showed that the HGD was clustered separately from the Asian wild ass clade. Therefore, these results indicate Africa as the most probable location for HGD domestication (Figure 6).

## 4. Discussion

Mitochondrial DNA is a powerful and widely used molecular marker for the estimation of a population’s phylogenetic relationships. It plays an important role in phylogenetic studies, comparative and evolutionary genomics, and molecular evolutionary analyses owing to its various advantageous characteristics, such as maternal inheritance, lack of recombination, and accelerated nucleotide substitution rates compared with those of the nuclear DNA [32,33]. The mtDNA D-loop is an extremely variable region characterized by fast evolution and a non-coding function, with the largest mutation rate in the whole mtDNA [34]. The degree of genetic diversity reflects the strength of biological evolution and evolutionary adaptation potential. To date, some studies have reported the complete mitochondrial genome, phylogeny, and maternal origin of the donkey using the full mitogenome and D-loop region [11,15,16,35]. To elucidate the genetic resource, we reported the sequence of the whole mitogenome, genetic diversity, and maternal origin of the HGD.

In this study, we used next-generation sequencing to reconstruct the complete mitochondrial genome of the HGD, and PCR to amplify the most variable region in the mtDNA D-loop. The results showed that the length of the full HGD mitogenome is 16,680 bp, of which the D-loop region is 1216 bp. As reported in previous genetic studies on donkeys [15,36], the D-loop region of ancestral mtDNA supplies adequate information to assess the genetic variation, evolutionary relationships, and matrilineal genetic origins. We detected 23 nucleotide polymorphic sites in the D-loop region sequences of the HGD mtDNA. Among the D-loop 418 bp fragments, polymorphic nucleotide sites accounted for 9.21%, (of which 94.44% were found to be transformed or transposed) and two insertion sites accounting for 5.56% [27]. Therefore, the frequency of transposition was much higher than that of transversion, which is consistent with our results. We found that the haplotype and nucleotide diversity were 0.87000 (Hd) and 0.02115 (Pi), respectively, which indicated the high genetic diversity of HGDs.

The traditional view on the origin and evolution of the Chinese donkey is that it originated from the African wild ass following domestication. Consistent with our study, Lei et al. [12] found that there are two mitochondrial origins for the African wild ass, lineage Somali (Clade II) and lineage Nubian (Clade I), in Chinese domestic donkeys, and that Clade II was prevalent in Chinese domestic donkey breeds. Our findings provide valuable insight into the evolutionary relationships within Equidae and can be useful for improving our understanding of equid phylogenetics.

## 5. Conclusions

The present study revealed a high mtDNA diversity in the D-loop region of HGDs, as indicated by the detection of 11 haplotypes. We confirmed that the Chinese donkey originated from the African wild ass following domestication, consistent with previous research. Moreover, we excluded the Asian wild ass as an ancestor of HGDs. These results can be used to inform future HGD phylogenetic studies and provide insights into the evolution of genomes.

## Figures and Tables

**Table 1:** Summary of the Huaibei grey donkey mitogenome.

Gene	Position		Length/bp	Strand	Anticodon	Codon	
	From	To				Start	Stop
tRNA-Phe	1	71	71	H	GAA		
12S rRNA	72	1046	975	H			
tRNA-Val	1046	1112	67	H	UAC		
16S rRNA	1113	2692	1580	H			
tRNA-Leu	2693	2767	75	H	UAA		
ND1	2770	3726	957	H		ATG	TAA
tRNA-Ile	3726	3794	69	H	GAU		
tRNA-Gln	3792	3864	73	L	UUG		
tRNA-Met	3867	3935	69	H	CAU		
ND2	3936	4976	1041	H		ATA	TAG
tRNA-Trp	4975	5043	69	H	UCA		
tRNA-Ala	5049	5117	69	L	UGC		
tRNA-Asn	5119	5191	73	L	GUU		
tRNA-Cys	5224	5289	66	L	GCA		
tRNA-Tyr	5290	5356	67	L	GUA		
COX1	5358	6902	1545	H		ATG	TAA
tRNA-Ser	6900	6968	69	L	UGA		
tRNA-Asp	6977	7043	67	H	GUC		
COX2	7045	7728	684	H		ATG	TAA
tRNA-Lys	7732	7800	69	H	UUU		
ATP8	7802	8005	204	H		ATG	TAA
ATP6	7963	8643	681	H		ATG	TAA
COX3	8643	9427	785	H		ATG	TAG
tRNA-Gly	9427	9496	70	H	UCC		
ND3	9497	9843	347	H		ATA	TAG
tRNA-Arg	9844	9912	69	H	UCG		
ND4L	9914	10,210	297	H		ATG	TAA
ND4	10,204	11,581	1378	H		ATG	AGA
tRNA-His	11,582	11,650	69	H	GUG		
tRNA-Ser	11,651	11,710	60	H	UGA		
tRNA-Leu	11,712	11,781	70	H	UAG		
ND5	11,773	13,602	1830	H		ATA	TAA
ND6	13,586	14,110	525	L		ATG	TAA
tRNA-Glu	14,114	14,182	69	L	UUC		
CYTB	14,187	15,326	1140	H		ATG	AGA
tRNA-Thr	15,327	15,398	72	H	UGU		
tRNA-Pro	15,400	15,465	66	L	UGG		
D-loop	15,466	16,681	1216	H			

Note: H: high strand, L: light strand.

**Table 2:** Composition and skew values in different regions of mitochondrial genome of Huaibei grey donkey.

Gene/Region	T(%)	C(%)	A(%)	G(%)	A+T(%)	AT-Skew	GC-Skew
tRNA	27.18	22.69	34.50	15.63	61.68	0.1187	−0.1842
rRNA	21.44	24.62	36.41	17.54	57.85	0.2588	−0.1679
ND1	27.38	30.20	31.14	11.29	58.52	0.0643	−0.4558
ND2	24.78	31.32	34.77	9.13	59.55	0.1678	−0.5486
ND3	26.86	28.86	31.14	13.14	58.00	0.0738	−0.3743
ND4	25.91	31.28	31.71	11.10	57.62	0.1007	−0.4762
ND5	25.63	32.08	31.69	10.60	57.32	0.1057	−0.5033
ND6	19.62	31.62	41.14	7.62	60.76	0.3542	−0.6116
ND4L	28.96	28.62	29.29	13.13	58.25	0.0057	−0.3710
COX1	28.61	27.38	28.03	15.99	56.64	−0.0102	−0.2626
COX2	25.58	28.07	32.46	13.89	58.04	0.1185	−0.3379
COX3	26.62	30.70	27.13	15.54	53.75	0.0095	−0.3279
ATP6	27.61	29.66	31.72	11.01	59.33	0.0693	−0.4586
ATP8	27.94	25.00	37.75	9.31	65.69	0.1493	−0.4573
CYTB	26.32	32.46	28.60	12.63	54.92	0.0415	−0.4398
D-loop	21.65	34.90	30.37	13.09	52.02	0.1676	−0.4545
Overall	25.59	28.88	32.31	13.22	57.90	0.1161	−0.3720

**Table 3:** Codon number and relative synonymous codon usage (RSCU) of Huaibei grey donkey mitochondrial DNA.

Codon	Count	RSCU	Codon	Count	RSCU	Codon	Count	RSCU	Codon	Count	RSCU
UUU(F)	47	0.65	UCU(S)	65	1.01	UAU(Y)	73	0.94	UGU(C)	9	0.82
UUC(F)	98	1.35	UCC(S)	108	1.67	UAC(Y)	82	1.06	UGC(C)	13	1.18
UUA(L)	73	1.13	UCA(S)	126	1.95	UAA(*)	63	1.06	UGA(*)	64	1.07
UUG(L)	17	0.26	UCG(S)	34	0.53	UAG(*)	52	0.87	UGG(W)	9	1
CUU(L)	53	0.82	CCU(P)	75	1.08	CAU(H)	72	0.98	CGU(R)	9	0.71
CUC(L)	75	1.16	CCC(P)	84	1.21	CAC(H)	75	1.02	CGC(R)	14	1.11
CUA(L)	137	2.12	CCA(P)	110	1.58	CAA(Q)	76	1.27	CGA(R)	21	1.66
CUG(L)	32	0.5	CCG(P)	9	0.13	CAG(Q)	44	0.73	CGG(R)	10	0.79
AUU(I)	62	0.65	ACU(T)	77	1.04	AAU(N)	62	0.89	AGU(S)	14	0.22
AUC(I)	125	1.3	ACC(T)	80	1.08	AAC(N)	77	1.11	AGC(S)	40	0.62
AUA(I)	101	1.05	ACA(T)	110	1.49	AAA(K)	90	1.75	AGA(R)	10	0.79
AUG(M)	36	1	ACG(T)	29	0.39	AAG(K)	13	0.25	AGG(R)	12	0.95
GUU(V)	22	0.86	GCU(A)	46	1.07	GAU(D)	31	0.93	GGU(G)	14	0.5
GUC(V)	28	1.1	GCC(A)	65	1.51	GAC(D)	36	1.07	GGC(G)	36	1.27
GUA(V)	38	1.49	GCA(A)	53	1.23	GAA(E)	64	1.56	GGA(G)	52	1.84
GUG(V)	14	0.55	GCG(A)	8	0.19	GAG(E)	18	0.44	GGG(G)	11	0.39

Note: * represents stop codon.

**Table 4:** Haplotypes and polymorphic sites of the Huaibei Grey donkey mitogenome.

Haplotypes	Haplogroup	Localization of Polymorphic Sites																						
		66	72	85	151	162	172	174	180	181	202	203	226	227	234	244	280	297	352	383	388	402	403	404
NC_001788.1		G	C	T	A	A	A	A	C	A	T	A	G	A	C	A	C	A	T	C	C	C	G	G
H1	Clade II	A	T	C	G	G	*	*	T	G	C	G	A	*	T	G	T	*	C	T	T	T	A	A
H2	Clade I	*	*	*	*	*	*	*	*	G	*	*	*	*	*	*	*	T	*	*	*	*	A	*
H3	Clade II	A	T	C	G	*	*	*	T	G	*	*	A	G	T	G	T	*	C	T	*	T	A	A
H4	Clade II	A	T	C	G	G	*	*	T	G	*	*	A	*	T	G	T	*	C	T	T	T	A	A
H5	Clade I	*	*	*	*	*	*	*	*	G	*	*	*	*	*	*	*	*	*	*	*	*	A	*
H6	Clade II	A	T	C	G	G	*	*	T	G	*	*	A	G	T	G	T	*	C	T	*	T	A	A
H7	Clade II	A	T	C	G	*	*	*	T	G	*	*	A	*	T	G	T	*	C	T	T	T	A	A
H8	Clade II	A	T	C	G	G	*	G	T	G	*	*	A	G	T	G	T	*	C	T	*	T	A	A
H9	Clade I	*	*	*	*	*	*	*	*	G	*	*	*	*	*	*	*	*	*	*	*	*	*	*
H10	Clade II	A	T	C	G	G	G	*	T	G	*	*	A	*	T	G	T	*	C	T	T	T	A	A
H11	Clade I	*	*	*	*	*	*	*	*	*	*	*	*	*	*	*	*	*	*	*	*	*	A	*

* represents identical nucleotides to the reference sequence (NC_001788.1).

**Table 5:** GenBank accession numbers of the 60 donkey mtDNA D-loop sequences used in the present study.

Country	Number	GenBank Accession Nos.	Reference
Egypt	15	MG656081.1~MG656095.1	[26]
Ethiopia	15	MG656111.1~MG656125.1	[26]
Turkey	30	MH683672.1~MH683701.1	[15]

## Data Availability

They will be provided during review.
